# A decade of reconstructive surgery: outcome and perspectives of free tissue transfer in the head and neck. Experience of a single center institution

**DOI:** 10.1007/s10006-020-00838-7

**Published:** 2020-03-20

**Authors:** Steffen Spoerl, Shlomo Schoedel, Gerrit Spanier, Karolina Mueller, Johannes K. Meier, Torsten E. Reichert, Tobias Ettl

**Affiliations:** 1 Department of Cranio- and Maxillofacial Surgery, Hospital of the University of Regensburg, Franz-Josef-Strauß-Allee 11, 93053 Regensburg, Germany; 2 Centre for Clinical Studies, Hospital of the University of Regensburg, Franz-Josef-Strauß-Allee 11, 93053 Regensburg, Germany

**Keywords:** Flap, Microvascular, Head and neck, Reconstruction, Outcome

## Abstract

**Purpose:**

Free flaps have become the standard option in reconstructive surgery of the head and neck. Even though many authors have outlined the reliability of free transplants, there is an ongoing discussion about treatment options for patients bearing particular risks as previous irradiation treatment. In this analysis, we aim to address these patients with particular risk profiles by comparing different flap entity outcome parameters.

**Methods:**

We retrospectively analyzed a cohort of 494 patients who underwent flap surgery between 2009 and 2018 in our department. Focusing on free microvascular transplants, we additionally analyzed the pectoralis major myocutaneous flap as the most frequently used vascular pedicled flap. Data analysis was performed by uni- and multivariate statistics.

**Results:**

Overall flap success rate was 90%, with the radial forearm flap occurring to be most reliable (93%) in head and neck reconstruction. Previous radiation therapy (RT) and intraoperative revision of vascular anastomosis during primary surgery significantly resulted in impaired transplant outcome with a success rate of 91.8% (no RT) vs. 83.7% (RT), respectively. There was a negative linear correlation between incision to suture time and number of flaps per year (*R*^2^ = 0.67).

**Conclusions:**

Preoperative radiation therapy and intraoperative revision of anastomosis significantly impair outcome of microvascular flaps in the head and neck and oral cavity, whereas patient’s age is not a predictor of flap failure. Increasing case number and experience reduces time of flap surgery as well as rate of complications and flap failure.

## Introduction

Even in times of microvascular surgery, large defects in the oral and maxillofacial area are challenging and represent one of the most difficult parts in head and neck surgery. After introduction of microsurgery-based free tissue transfer in the 1970s, tremendous progress has been made to improve operative techniques and perioperative patient management. Regardless of distinct flap entities, success rates of microvascular flaps in the head and neck region are reported to range over 90% [1]. Regarding outcome parameters, single center analysis of microvascular flap outcome in the head and neck region has emphasized the importance of the surgeon’s experience, operative techniques as well as postoperative flap monitoring in terms of flap success and avoiding perioperative complications [2]. Especially in the last years, a more detailed evaluation on potential pre-, peri- as well as postoperative risk factors in microvascular flap surgery in the head and neck region had been made: Previous radiation therapy (RT) in the head and neck had recently been underlined by numerous authors to act as one of the most important preoperative risk factors for flap loss and impaired wound healing [3]. To date, there is only limited literature available about treatment options for large defects in the head and neck region for irradiated patients.

We therefore performed a 10-year monocentric retrospective study for microvascular flaps in the head and neck region from 2009 to 2018. Besides evaluating the impact of previous RT in outcome parameters of microvascular flaps, we evaluated transplant loss for distinct flap entities and addressed previously described risk factors for flap outcome like use of a venous coupler, perioperative antithrombotic therapy, and patient’s age. Beyond that, we assessed whether intraoperative revision of microvascular anastomosis might have deleterious effects on transplant survival.

## Materials and methods

This retrospective monocentric analysis was conducted in the Department of Cranio- and Maxillofacial Surgery, Hospital of the University of Regensburg, Germany. Overall, 494 cases of free and distant flaps, transplanted into the oral cavity and head and neck region, were evaluated. Data was obtained by reviewing digital and written patient records. Basic patient and treatment data is shown in Table 1. The main diagnosis requiring reconstructive flap surgery was tumor (88%), another 9% of flaps were performed due to osteoradionecrosis. Median age was 62 years, with the oldest patient in this cohort being 92 years. Most of the patients were male (64%); nicotine abuse was diagnosed in 62% of patients. Preoperative RT in the head and neck region was performed in 23% of cases. Flap success was defined as a functional transplant without any signs for transplant loss up to 6 weeks after surgery. Transplant complications were divided into venous, arterial, and other reasons, whereas other reasons entailed hematomata and combined arterial and venous complications. Donor site morbidity entailed impaired wound healing, seromata, hematomata as well as severe consequences like compartment syndrome.

**Table 1:** Clinicopathological characteristics

Patient characteristics	*N*	Percentage
Number of flaps 2009–2018	494	
Gender		
Male	315	64
Female	177	36
Alcohol abuse	221	45
Nicotin abuse	306	62
Median days at ICU (0–47 days)	2	
Median age at flap surgery in years (20–92 years)	62	
Diagnosis at surgery		
Tumor	433	88
Osteoradionecrosis	44	9
Medication-related osteonecrosis	5	1
Osteomyelitis of other origin	10	2
Other	2	< 1
Site of reconstruction		
Mandible	170	34
Maxilla, palate	50	10
Floor of mouth, tongue	169	34
Intermaxillary	61	12
Extraoral	44	9
Previous radiotherapy in the head and neck	129	26
Previous surgery in the head and neck	236	48
Venous coupling of anastomosis	170	34
Tracheostomy	235	48
Flap success	443	90
Postoperative revision of anastomosis	51	10
Impaired wound heeling	149	30
Flap complication	97	20
Venous	39	8
Arterial	10	2
Other (e.g., venous/arterial, bleeding, hematoma)	48	10

For statistical analysis SPSS (v. 25) was used, a *p* value ≤ 0.05 was defined as statistically significant. For univariate analysis, chi-square and Fisher’s exact test were used to compare different groups of outcome parameters with and without the presence of distinct pre- and perioperative risk factors. Linear correlation was applied to assess whether the number of performed flap surgeries has an impact on the incision to suture time. Binary logistic regression (enter method) was used to evaluate the impact of various pre- and perioperative risk factors (previous RT, revision of anastomosis intra primary surgery, dosage of heparin, sex, age, nicotine abuse as well as use of a venous coupler) on microvascular transplant survival.

## Results

For this retrospective analysis, we included 494 patients receiving free or distant flaps, 451 flaps were free microvascular transplants. Most common performed flaps were the radial forearm flap (*n* = 230), the free fibula flap (*n* = 121), and the anterolateral thigh flap (ALT) (*n* = 51). The pectoralis major flap (*n* = 40) represented the most frequently used vascular pedicled transplant (Table 2).

**Table 2:** Outcome parameters in different flap entities

Flap entity	*N*	Flap success (%)	Postoperative revision of anastomosis (%)	Impaired wound healing (%)
Radial forearm flap	230	93	10	29
Anterolateral thigh flap	51	82	18	35
Free upper arm flap	5	100	0	0
Latissimus dorsi flap	25	92	*	24
Free fibula flap	121	88	14	34
Deep circumflex iliac artery flap	13	85	8	38
Scapular flap	4	75	0	25
Pectoralis major myocutaneous flap	40	88	*	28
Submental island flap	5	80	*	20

*Flaps were (partly) applied as vascular pedicled transplants

**Fig. 1:**
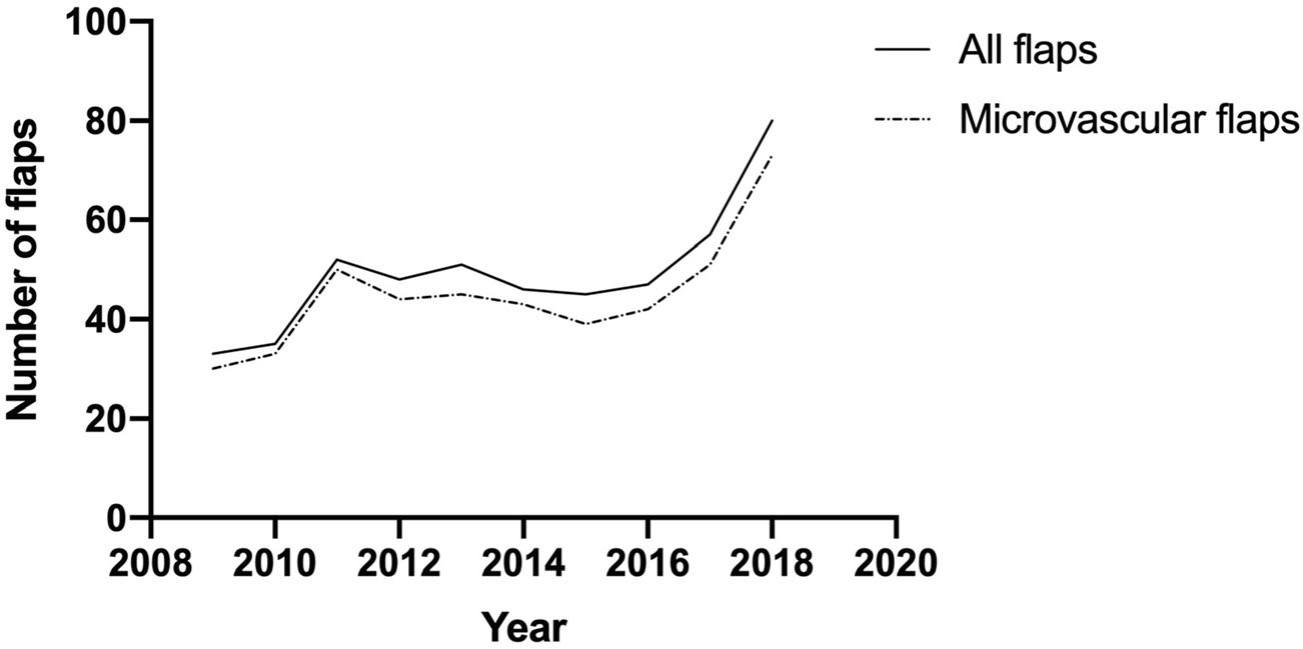
Evolution of flap surgery from 2009 until 2018 in a monocenter institution

During the 10-year timeframe of head and neck free tissue transplant surgeries in our institution, the number of microvascular flaps per year could be shown to increase from 30 cases in 2009 to 73 in 2018 (Fig. 1).

Overall flap success was 90%, ranging from 75 (scapular flap) to 100% (free upper arm flap). The radial forearm flap was reported to be successful in 93% of all cases (Table 2). With regard to the last years, overall success rate notably increased from 88 (2009–2016) to 93% (2017, 2018) (data not shown). This was particularly obvious for the ALT where the success rates raised from just 79% at the beginning to 92% during the last years (2017, 2018). Postoperative revision of anastomosis was observed in 10% of radial forearm flaps while the free fibula flap and the ALT were ranging at 14% and 18%, respectively (Table 2). Regarding prevalence of impaired wound healing in clinically relevant flap entities, wound healing abnormalities were documented in 30% of all flaps with lowest rates of 24% in latissimus dorsi transplants and highest (38%) in deep circumflex iliac artery flaps which were all harvested as muscle flaps without a skin island (Table 2).

**Fig. 2:**
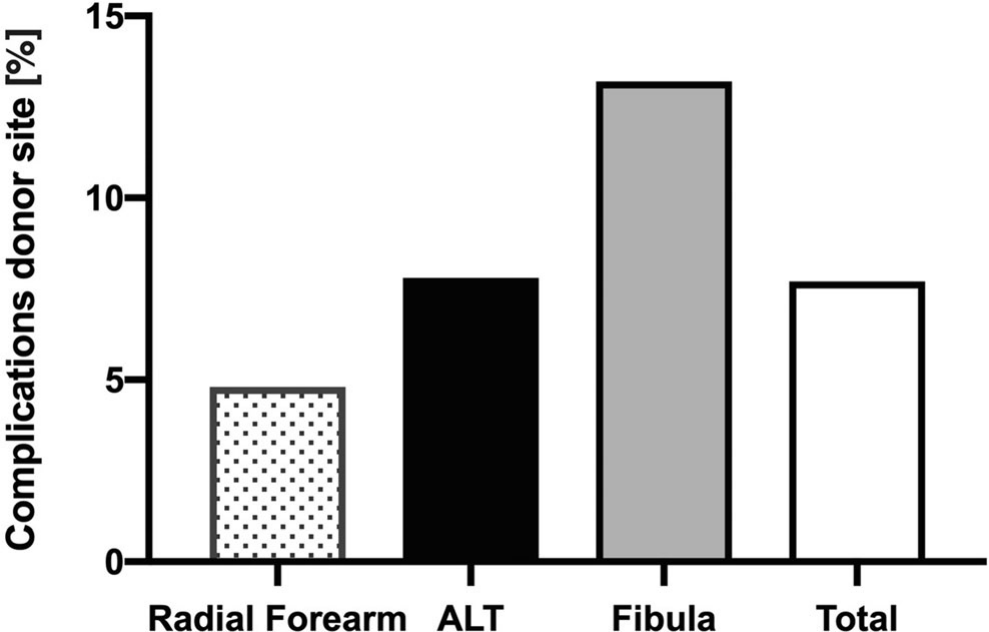
Complication rates of donor site in different microvascular flap entities, *p* = 0.019

Furthermore we investigated differences in prevalence of donor site complications within the most commonly used microvascular flap entities. Here peri- and postoperative complications occurred in under 5% of radial forearm transplants, compared with the significantly increased rates for the free fibula flap and the ALT (Fig. 2).

**Fig. 3:**
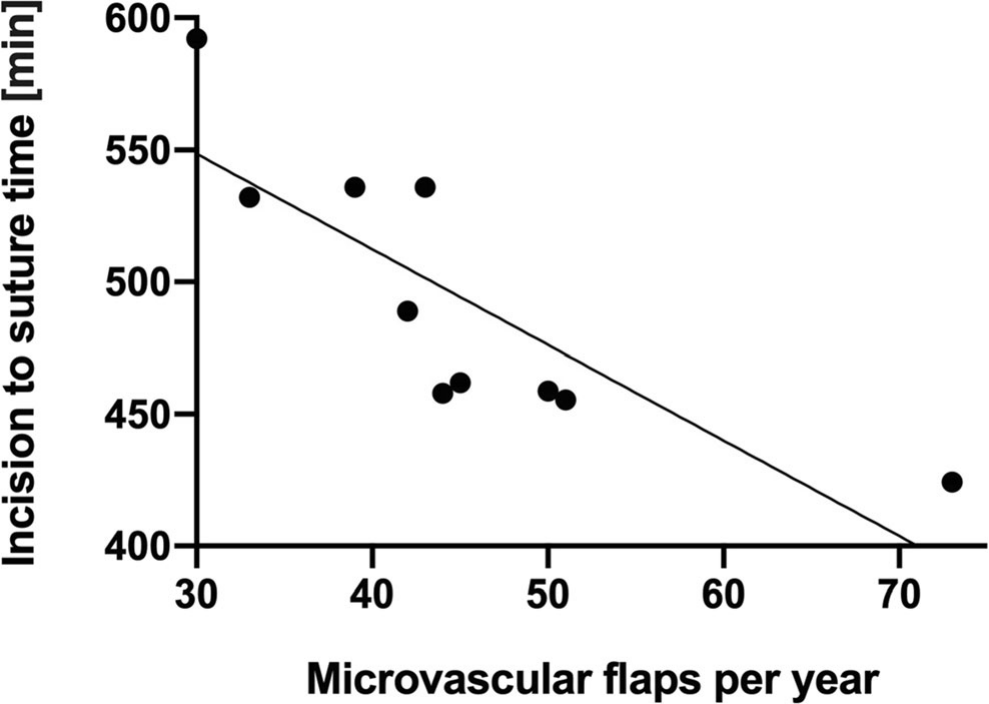
Correlation analysis between number of performed flaps per year and incision to suture time; *p* = 0.000, *r* = − 0.18

Having reported a profound increase in flap surgeries per year, we investigated if the number of accomplished flaps per year on the one hand and the incision to suture time on the other hand might correlate. Figure 3 therefore shows a negative linear correlation between total surgery time and number of flaps per year with *R*^2^ = 0.67.

**Fig. 4:**
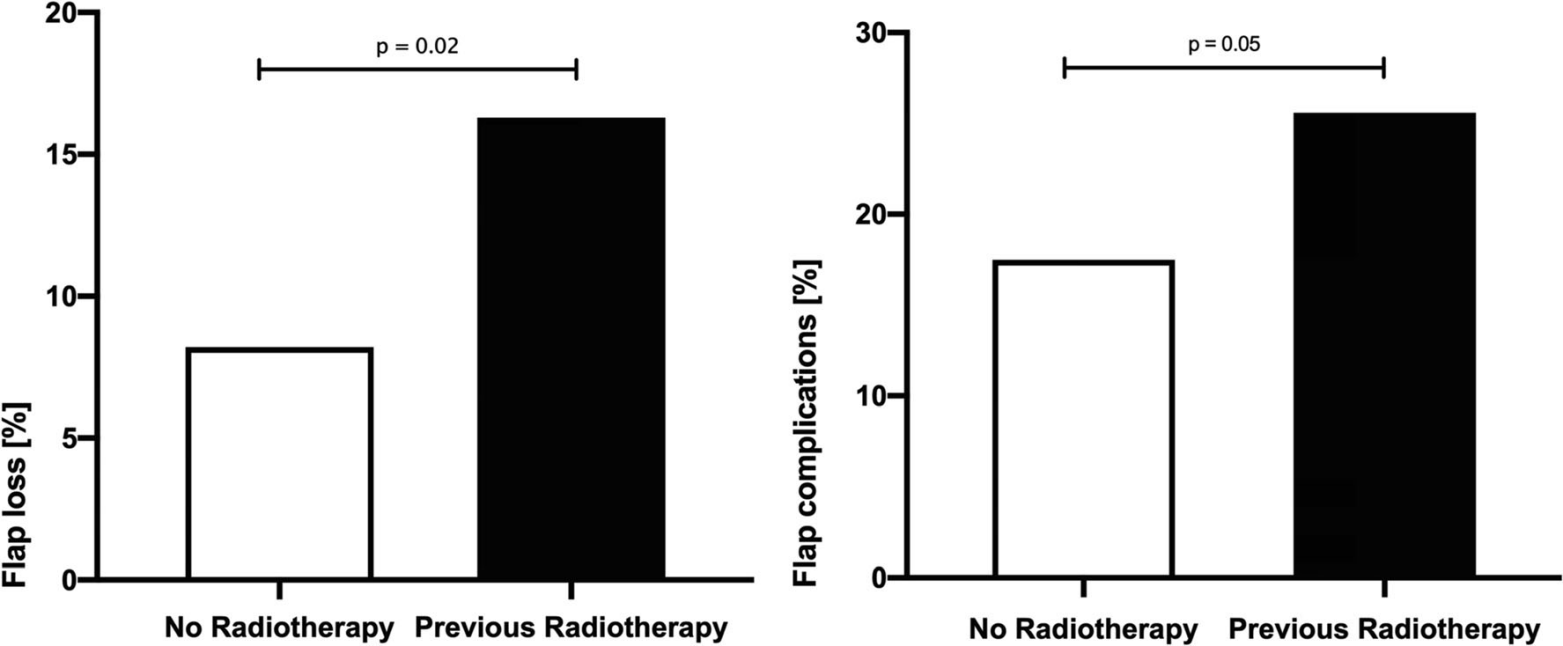
Impact of preoperative radiotherapy of the head and neck region on flap loss and flap complications

**Fig. 5:**
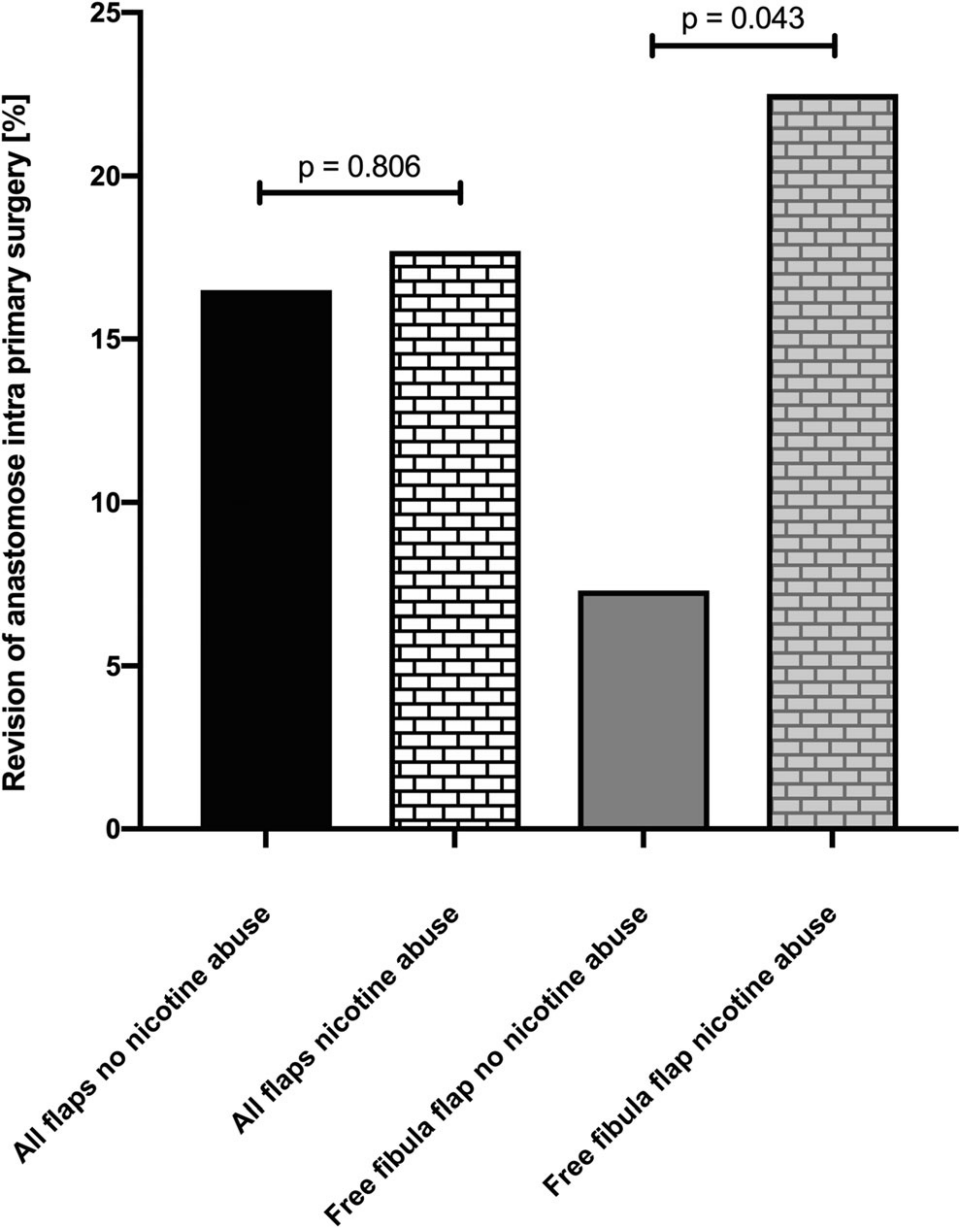
Impact of smoking on revision of anastomosis during primary surgery within all flaps and the free fibula flap

Questioning if RT prior to primary surgery might alter transplant outcome, we performed a univariate analysis (Fisher exact test), revealing preoperative RT in the head and neck region to significantly increase flap loss as well as complication rates in comparison with unirradiated patients. Preoperative RT doubled the risk of transplant failure with an overall success rate of 92% in non-irradiated and 84% in patients having a history of preoperative irradiation of the head and neck (Fig. 4). Furthermore, we aimed to evaluate the impact of smoking on flap success. Hereby no significant differences could be seen for the totality of all flaps, whereas focusing on free fibula flaps solely revealed a significantly elevated incidence of anastomosis revision during primary surgery in the group of smokers (Fig. 5).

The impact of distinct pre- and perioperative factors on flap loss was assessed with binary logistic regression. Analogous to univariate analysis, preoperative RT significantly increased the risk of transplant loss also in multivariate analysis (Table 3). With an OR of 2.6, the risk of flap loss was also significantly increased if intraoperative revision of anastomosis was required during primary surgery (Table 3).

**Table 3:** Binary logistic regression of different risk factors on flap loss

Factor	Sig.	Odds ratio	95% confidence interval, lowest value	95% confidence interval, highest value
Previous radiotherapy	0.007	2.372	1.271	4.424
Revision of anastomosis during primary surgery	0.005	2.604	1.338	5.068
Use of heparin: prophylactic vs. therapeutic doses	0.480	0.779	0.389	1.559
Sex	0.371	1.338	0.708	2.529
Age at surgery	0.442	0.991	0.967	1.015
Smoking	0.949	1.021	0.534	1.955
Use of a venous coupler	0.211	0.650	0.331	1.276

Model summary: *x*^2^ = 18.522, *p* = 0.018, Nagelkerke’s *R*^2^ = 0.077

Age, sex, nicotine abuse, use of venous coupling as well as different doses of perioperatively administered antithrombotic medication did not affect the risk of transplant failure in our cohort (Table 3).

## Discussion

The current retrospective investigation shows the development of experience in free flap reconstructive surgery in a single maxillofacial unit during a period of 10 years. In this period, the radial forearm flap (RFF) and the free fibular flap (FFF) emerged as main workhorse flaps for save and reliable reconstruction of major hard and soft tissue defects in the head and neck area. During the last few years, the anterolateral thigh flap (ALT) has been established as third workhorse and is also increasingly performed in our clinic due to its variable options in size and tissue components [4]. It has to be mentioned that the ALT is not as standardized as the RFF and that there is an individual learning curve of the surgeon performing the flap due to its varying course of the perforators [4]. Also in our institution, success rates for the ALT were raised from just 79% at the beginning to 92% during the last years (2017, 2018). In this context, perforator and “super” perforator flaps represent the most current development of free flap reconstruction with minimized donor site morbidity and favorable success rates [5]. However, these flaps are by far not as standardized as a radial forearm flap—which can easily be learned during residency—and often need a reconstructive back-up plan in case of insufficient perforator. With view to the current literature, it has to be mentioned that our overall flap success rate of 90% is slightly lower than reported survival rates of 95% and more in high volume centers [2]. The aim of this presentation, however, is not to compete with these huge institutions that are performing microsurgery for decades in contrast to our institution where these complex surgeries just started some years ago. Nevertheless, regarding the last 2 years of the survey, a success rate of 93% seems rather acceptable.

In our department, with increasing number of free flaps incision to suture time was markedly reduced by one-third during the 10-year period which shows the increase of experience and routine during that period. A further reason for the time reduction is a consequent two-team approach in free flap surgery. Reduction of time in major reconstructive surgery not only saves personal energy and motivation but also decreases the risk of postoperative complications as wound infection, dehiscences, hematoma, and seroma [6].

Even though free tissue transfer has become routine in reconstructive surgery of the head and neck, there is little information about the outcome parameters of different microvascular as well as vascular pedicled flap entities. In the current investigation, the pectoralis major myocutaneous flap (PMMF) was evaluated as the most commonly performed vascular pedicled flap and came up with a slightly lower success rate of 88%. However, this option was mainly chosen due to limitations of microvascular free tissue transfer and was selected as a compromise solution in high-risk patients after previous surgeries and irradiation. According to Liu et al., the PMMF is ideally applied for patients with free flap failure, previous RT, or for selected patients not tolerating prolonged surgery [7]. Despite the PMM flap’s complication rate, being characterized by a relevant number of partial necrosis and impaired wound healing [8, 9], it remains a main option for reconstructive salvage surgery in selected patients. Alternatively—in vessel depleted necks or compromised flaps—studies on temporarily free flap supply by extracorporeal perfusion are currently performed which may present an option in the future although the required resources are tremendous [10].

Preoperative RT has been linked to deleterious outcome of free tissue transfers in the head and neck region [11]. However, preoperative RT is commonly present in patients, receiving distant flap surgery in the head and neck and oral cavity region: 23% of patients in this retrospective cohort had a history of RT, mainly because of primary radiochemotherapy or adjuvant treatment of head and neck tumors. The need for a free tissue transfer was commonly due to tumor relapse, secondary carcinoma, or osteoradionecrosis. For not irradiated patients, successful free tissue transfer rates in the head and neck are in accordance with our findings and stated to range over 90% [12]. Especially for higher RT dose ≥ 60 Gy, which are commonly applied for head and neck squamous cell carcinoma (HNSCC) - patients [13], flap failure rates up to 21% and increased local complications like fistula formation and wound infection were described [12]. In this study, univariate and multivariate analyses underlined the unfavorable effect of preoperative RT prior to free tissue transfer in the head and neck.

Regarding multivariate analysis, we were able to show that revision of anastomosis within primary surgery resulted in an enhanced rate of flap loss and therefore represents a negative individual predictor for flap success. Even though this effect—at least from surgeon’s point of view—is somehow expected, our extensive analysis was the first one to confirm this hypothesis.

Atherosclerosis is supposed to aggravate microvascular flap surgery. In a comprehensive study population, full clinical picture of peripheral arterial disease (PAD) or earlier stages of pulse abnormalities were diagnosed in over 20%, affecting mostly the lower extremity [14]. And also from the own surgical experience, profound atherosclerotic signs were repeatedly found in the intima of fibular vessels. In this context, nicotine—as major reason for atherosclerosis and PAD—was identified as potent risk factor for intraoperative revision of anastomosis when performing free fibular transplants.

Having addressed the topic of RT prior to flap surgery, one particular group of patients is frequently not adequately addressed in retrospective analysis: the group of elderly patients. An increasing number of comorbidities as diabetes, atherosclerosis, arterial hypertension as well as prolonged surgery times raise the question about the ideal approach for reconstructive surgery, especially in older HNSCC patients [3]. Although numerous definitions for “the elderly patient” can be found in literature, we defined the group of elderly patients by the chronological age, with a cutoff age of 65 years. Having reported no significant difference in flap success in different patient ages, there is still an ongoing debate on complication rates for both groups [3, 15, 16]. In accordance with previous publications, multivariate analysis of flap success in elderly patients revealed no significant increase in flap losses in our cohort. In this regard, it might be adequate to describe free tissue transfer as a safe and feasible method of reconstructive surgery in patients independently from age, in the full knowledge that a successful reconstruction naturally requires a certain level of physical performance.

Of course this study has several limitations due to its retrospective character. For example, we are lacking information about the duration of flap ischemia during primary surgery, which is definitely regarded as a relevant risk factor in terms of transplant survival and flap complications. Nevertheless, we were able to address distinct influence factors on outcome of flap surgery in the head and neck region based on written and digital patient records, knowing that subjective assessments might affect results of this publication.

## Conclusion

During the last 25 years, free flap surgery has evolved as first reconstructive option for advanced oropharyngeal and facial defects. In the current study, we present the development of flap surgery in a tertiary maxillofacial unit during one decade. With increasing number of flap cases, institutional experience rises, resulting in superior success rates while surgery time was markedly reduced. Investigation of patient and treatment specific factors influencing outcome of flap surgery shows that prior RT in the head and neck region as well as intraoperative revision of anastomosis significantly increases transplant loss and complication rates and represents negative predictors of flap success. Overall, free flap reconstruction of head and neck defects represents state of the art also in elder patients.
